# Development of domain-specific epistemological beliefs of physiotherapists: a longitudinal study

**DOI:** 10.1186/s12909-019-1844-z

**Published:** 2019-11-01

**Authors:** Martina Bientzle, Ulrike Cress, Joachim Kimmerle

**Affiliations:** 1 Knowledge Construction Lab, Leibniz-Institut für Wissensmedien (IWM), Schleichstr. 6, 72076 Tuebingen, Germany; 2 Department of Psychology, University of Tuebingen, Schleichstr. 4, 72076 Tuebingen, Germany

**Keywords:** Epistemological beliefs, Therapeutic health concepts, Longitudinal study, Development

## Abstract

**Background:**

Epistemological beliefs (EBs) and therapeutic health concepts are two important factors of influence that affect how healthcare professionals process treatment-relevant information. A previous investigation compared physiotherapy students’ EBs and therapeutic health concepts with those of professionals in a cross-sectional study. That study design, however, did not allow for any conclusions about the temporal development of these concepts. This shortcoming has been addressed in the study presented here, which aimed to assess that temporal development.

**Methods:**

In a longitudinal study, physiotherapists filled in a questionnaire that measured their personal EBs about physiotherapy and about medicine, as well as their biomedical and biopsychosocial therapeutic health concepts. The participants were first examined during their medical training (December 2011). The follow-up measure was about 3 years later when the participants had become professional physiotherapists (January 2015). The development of their EBs was examined using paired sample t-tests and Wilcoxon signed-rank test.

**Results:**

The analysis was based on 41 participants who filled in the questionnaire at both time points and were working as physiotherapists at the time of the second measurement. There was a development of physiotherapy-related and a development of medicine-related EBs: Physiotherapy-related as well as medicine-related EBs were more sophisticated when physiotherapists had already entered the working world than during their physiotherapy training. Due to psychometric problems of the scales, the development of their therapeutic health concepts could not be analyzed.

**Conclusions:**

EBs are an important factor for (lifelong) learning. Physiotherapy-related and medicine-related EBs developed similarly in both domains. This is an indication that the temporal development of EBs is an expression of professionalization of healthcare personnel in their occupational field. The findings demonstrate that the development of EBs is not completed at the end of vocational training; it appears to be a development that continues even after the transition to professional life.

## Background

Health professionals must evaluate health-related information in the course of their daily work and take such information into consideration in their treatment decisions. Health-related information is often fragile and sometimes ambiguous [1–3]. Dealing with information in general and with health-related information in particular is influenced by people’s personal beliefs about the characteristics of scientific information. *Epistemological beliefs* (EBs) are one important factor that has an impact on how health professionals process medical information [4–6]. EBs “are fundamental assumptions about the nature of knowledge, the certainty of knowing, the criteria and justifications for knowing” [p.29, 7]. As Roex and Degryse [6] pointed out, EBs are especially important for assessing one’s own knowledge skills and learning strategies, and thus for making progress in acquiring medical knowledge. Acquiring knowledge is a lifelong process that consists of keeping up with the rapid advances in medical research. In the medical field [7], but also in other domains, such as mathematics [8] or reading comprehension [9], positive relationships between sophisticated EBs and learning success have frequently been observed (for an overview see [10]). EBs can be developed through problem-based learning settings and through arguing about different perspectives [11]. As a consequence of developing more sophisticated EBs, people tend to consider scientific knowledge to be more tentative, contextual, and subjective. Handling ill-structured problems [5] and dealing with patients with their individual medical histories and concerns are also relevant factors in the development of EBs [4] in medical contexts.

Previous research has shown that EBs can be regarded as domain-specific beliefs that also have a domain-general element [12, 13]. Students evaluate knowledge in science as more certain, for example, than knowledge in psychology [14]. They also assess knowledge in biology to be more tentative than knowledge in physics [15]. At the same time, however, advanced students in general have more sophisticated EBs than freshmen. As “mental structures were likely to be both context- and content-dependent” (p.202, [16]) the investigation of the development of EBs should take a discipline-specific perspective into account.

Another important factor that has an impact on how health professionals process health-related information is their own personal therapeutic health concept [17]. In today’s healthcare system, two therapeutic health concepts are relevant: a biomedical (*bm*) therapeutic health concept and a biopsychological (*bps*) therapeutic health concept. This relevance is reflected in the common occurrence of the International Classification of Diseases (ICD) [18] (based on the *bm* concept) and the International Classification of Functioning, Disability and Health (ICF) [19] (based on the *bps* concept). The widely used approach of patient-centeredness is closely linked to the *bps* concept [20, 21]. Health professionals’ personal therapeutic health concepts influence how they communicate with patients [22] and how they handle treatment-relevant information [23, 24]. As physiotherapists aim to promote patients’ participation and activity in their daily life, the *bps* concept is supposed to be more prevalent among physiotherapists than the *bm* concept [25, 26]. However, adopting this perspective is challenging for many physiotherapists [27], even though there is evidence that a *bps* approach can be more effective in treating patients than a *bm* approach [28]. It is conceivable that the development of therapeutic health concepts could be part of the professionalization process of heath personnel. So far, however, not much is known about health professionals’ temporal development of *bm* and *bps* concepts.

Epistemological beliefs and therapeutic health concepts are two important factors of influence that affect how health professionals process treatment-relevant information. A cross-sectional study found that EBs and therapeutic health concepts differed depending on the participants’ training status [29]. Professional physiotherapists showed more sophisticated physiotherapy-related and more sophisticated medicine-related EBs than physiotherapy students. It was also found that professional physiotherapists had a more pronounced *bm* concept than students. Moreover, professional physiotherapists had a more pronounced *bps* concept than first-year students. No conclusions can be drawn about the temporal development of the concepts from such a cross-sectional study. Thus, the present study aimed to examine the development of EBs and therapeutic health concepts over time with a longitudinal study design. Based on the previous considerations, the following hypotheses were stated:

Hypothesis 1: There will be a development of EBs:

- Physiotherapy-related EBs will be more sophisticated when physiotherapists have already entered the working world than during their physiotherapy training.
- Medicine-related EBs will be more sophisticated when physiotherapists have already entered the working world than during their physiotherapy training.

Hypothesis 2: There will be a development of the therapeutic health concept:

- The *bm* health concept will be more pronounced when physiotherapists have already entered the working world than during their physiotherapy training.
- The *bps* health concept will be more pronounced when physiotherapists have already entered the working world than during their physiotherapy training.

## Methods

### Participants

**Fig. 1:**
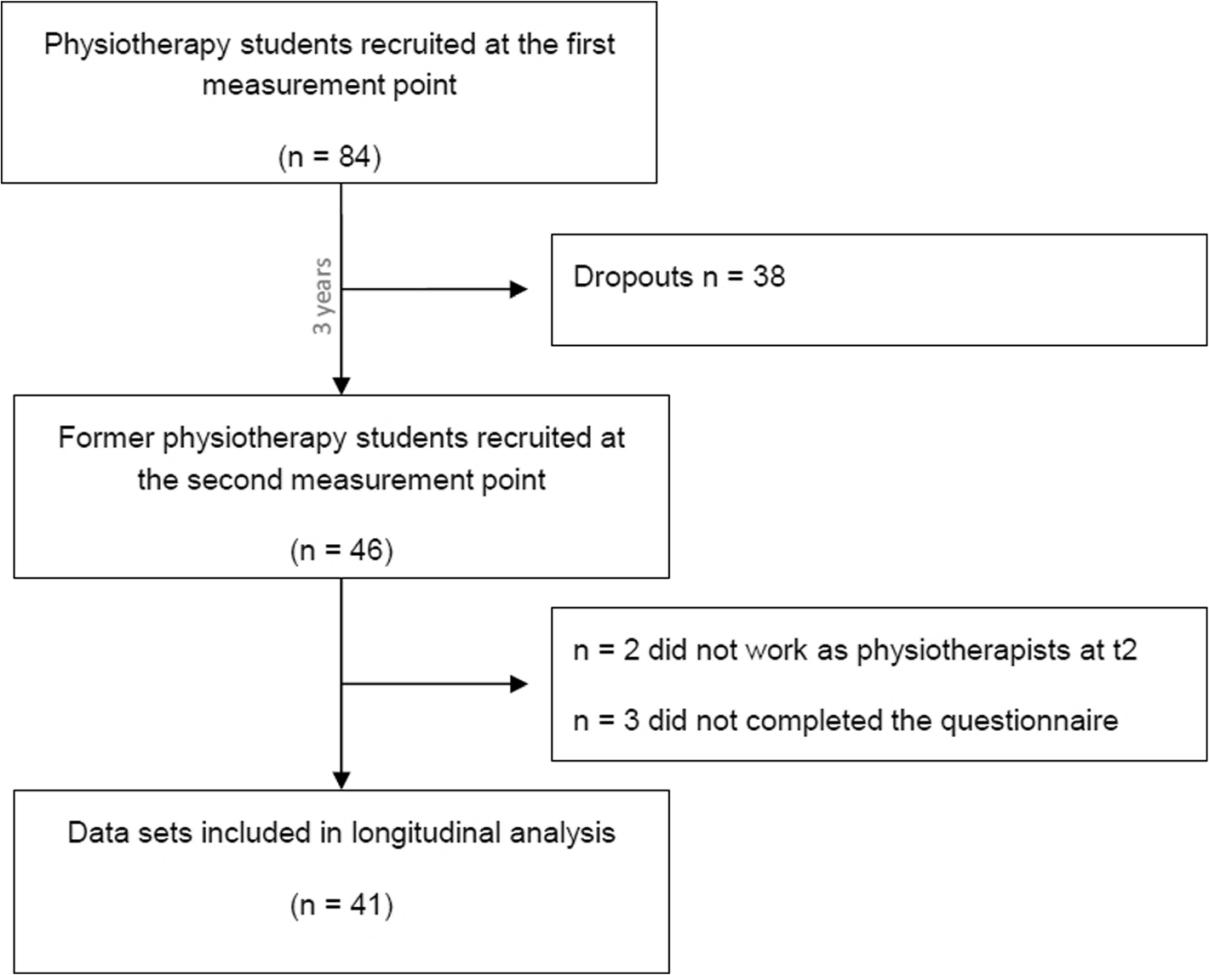
Flow diagram of study design

Eighty-four participants took part in this longitudinal study at the first measurement point (December 2011; t1). At that time, they were all students at a school of physiotherapy in Tübingen, Germany (PT Academy). Twenty-nine students were first-year students and 55 were advanced students (second and third year). At t1, 80 students (95%) were between 21 and 30 years old, only 4 students (5%) were between 31 and 40. Fifty-eight were women, 26 were men. Forty-six physiotherapists (55% of those who had participated in the first measurement) filled in the questionnaire at the second measurement point (January 2015; t2). At that time, they were working in different places (multiple answers were possible): Six were working in a hospital (four in an emergency hospital, two in a rehabilitation center), 37 were employed in a physiotherapy practice, two were self-employed in a physiotherapy practice, and three were working in the sports sector. Two of the participants were not working as physiotherapists at t2. The following analysis is based on those 41 participants who filled in the questionnaire at both measurement points and were working as physiotherapists at the time of t2 (see Fig. 1).

### Procedure

The data collection at t1 took place at the PT Academy. After informing the students about privacy protection, their right to terminate their participation at any time without any disadvantage, and about the general purpose of the study, they gave written informed consent. Then they were asked to fill in the questionnaire (see below).

The follow-up measurement was conducted online 3 years later. All participants who were students at the PT Academy at t1 were contacted by e-mail from the secretary’s office of the PT Academy. They were asked to fill in an online questionnaire. Participation took about 15 min and was compensated with the opportunity to take part in a lottery, where participants could win ten vouchers worth 15 Euros each. The datasets were linked by a code that the participants had created individually following a fixed procedure (the first letter of their mother’s first name, the first letter of their father’s first name, and their mother’s year of birth).

### Measurements

As described in the procedure of the cross-sectional analysis [29], the health-related EBs were measured with the Connotative Aspects of Epistemological Beliefs (CAEB) scale [30]. This scale was adapted to the domains of physiotherapy and medicine respectively. The participants assessed the knowledge in the field of physiotherapy and in medicine on seven-point semantic differential scales. Analogously to the cross-sectional study the CAEB-subscale *texture* was used*,* in which both domains were described with the same ten pairs of adjectives respectively. Sample items of this scale are “sorted – unsorted” or “absolute – relative”, whereby “unsorted” and “relative” represented sophisticated EBs (see Table 1).

**Table 1:** The texture subscale of the Connotative Aspects of Epistemological Beliefs (CAEB) scale

Knowledge in the domain of physiotherapy [or medicine respectively] is …			
1	objective	□-□-□-□-□-□-□	subjective
2	confirmable	□-□-□-□-□-□-□	unconfirmable
3	superficial	□-□-□-□-□-□-□	profound
4	exact	□-□-□-□-□-□-□	vague
5	absolute	□-□-□-□-□-□-□	relative
6	sorted	□-□-□-□-□-□-□	unsorted
7	precise	□-□-□-□-□-□-□	imprecise
8	definite	□-□-□-□-□-□-□	ambiguous
9^r^	negotiated	□-□-□-□-□-□-□	discovered
10	structured	□-□-□-□-□-□-□	unstructured

Seven-point semantic differential scales for measuring EBs about physiotherapy and medicine (texture subscale); reversely coded items are marked with a superscript ‘r’

The *bm* and *bps* health concepts were measured following the procedure presented previously [17, 29]. Participants rated the importance of five representative *bm* terms, such as “diagnosis”, and five characteristic *bps* concepts, such as “functionality”, on six-point Likert scales ranging from 1 (not important) to 6 (very important) (see Table 2).

**Table 2:** Therapeutic health concepts scale

How important are the following subjects for your therapeutic thinking and acting?	
1	Functionality
2*	Diagnosis
3*	Science
4*	Evidence-based methods
5	Limited activity of a patient
6*	Standardized tests
7	Limited participation of a patient (in the social environment)
8*	Medical guidelines
9	Mental health of a patient
10	Requirements of the patient’s everyday life

Six-point Likert scales for measuring the bm and bps concepts; bm items are marked with an asterisk (*)

### Statistical analysis

Data analysis was performed using IBM SPSS 20.0 for Windows [31]. Cronbach’s Alpha was calculated to determine internal consistency for all scales. All data are reported as means (M) ± standard deviations and median values. For testing the hypotheses, paired sample t-tests were applied. Paired sample t-tests were also applied for comparing EBs regarding physiotherapy to EBs regarding medicine at t1 and t2. As it is discussed whether t-tests should be used for Likert scales [32], Wilcoxon signed-rank tests and Mann-Whitney U tests were also calculated, as nonparametric test equivalents to the t-tests.

The level of significance was set at *P* < 0.050. Cohen’s d scores were calculated as effect sizes of mean differences.

### Ethics statement

This research was performed in accordance with the Declaration of Helsinki. The PT Academy’s administration provided ethical approval for the participation of its students (due to legal specifications, the school administration was responsible for checking and approving the participation of its students). Regarding the follow-up measurement, this study had full approval of the ethics committee of the Leibniz-Institut für Wissensmedien (approval number: LEK 2013/035). All participants took part voluntarily and anonymously.

## Results

### Health-related EBs

The internal consistency of the CAEB-subscale texture was acceptable in both domains (t1: α_phys_ = 0.62 α_med_ = 0.65; t2: α_phys_ = 0.66; α_med_ = 0.67).

Hypothesis 1a predicted a development of physiotherapy-related EBs. This assumption was supported by the data (see Table 3 for means and standard deviations). There were significant differences between the physiotherapy-related EBs at t1 and t2, t(40) = − 4.26, *P* < 0.001, d = 0.66 (Wilcoxon signed-rank test: z = − 3.50, *P* < 0.001).

**Table 3:** Health-related EBs

EBs Time of measurement	EBs (texture) regarding knowledge in physiotherapy	EBs (texture) regarding knowledge in medicine
First measurement	M = 3.37 ± 0.62	M = 3.09 ± 0.57
	Median = 3.40	Median = 3.10
Follow-up measurement	M = 3.82 ± 0.59	M = 3.60 ± 0.57
	Median = 3.80	Median = 3.50

Main effects of time of measurement on EBs regarding knowledge in physiotherapy and knowledge in medicine

Hypothesis 1b predicted a development of medicine-related EBs. This assumption was also supported by the data (see Table 3). Significant differences between the medicine-related EBs at t1 and t2 were found, t(40) = − 5.04, *P* < 0.001, d = 0.79 (Wilcoxon signed-rank test: z = − 4.15, *P* < 0.001).

In accordance with the cross-sectional study [29] an additional explorative analysis disclosed that first-year students showed greater development (M_Diff_ = 0.82 ± 0.56) than the advanced students (M_Diff_ = 0.39 ± 0.66) regarding medicine-related EBs, t(40) = 1.97, *P* = 0.028, d = 0.70 (Mann-Whitney-U-test: U = 105.00, *P* = 0.024).

Finally, like at t1, t(40) = 3.67, *P* < 0.001, d = 0.57 (Wilcoxon signed-rank test: z = − 3.40, *P* = 0.001), the EBs regarding physiotherapy were still more sophisticated than EBs regarding medicine at t2, t(40) = 2.50, *P* = 0.009, d = 0.39 (Wilcoxon signed-rank test: z = − 2.27, *P* = 0.023). The difference between physiotherapy- and medicine-related EBs were similar at both time points (Diff_t1_ = 0.28, SD = 0.49; Diff_t2_ = 0.21, SD = 0.56; t(40) = 0.64, *p* = .262).

### Therapeutic health concepts

The internal consistency of the subscale *bm* health concept was poor (t1: α = 0.54; t2: α = 0.63). The same was true of the *bps* heath concept scale (t1: α = 0.57; t2: α = 0.52). Due to this psychometric shortcoming, no analyses regarding therapeutic health concepts could be conducted.

## Discussion

The aim of this longitudinal study was to contribute to a better understanding of the temporal development of health-related EBs and therapeutic health concepts of physiotherapy students over a period of 3 years. A previous cross-sectional study found that the EBs differed between students and professionals [29]. The longitudinal findings support these earlier results in that they demonstrate that both physiotherapy-related and medicine-related EBs developed over time as people entered the working world. Former first-year students showed greater development of medicine-related EBs than former advanced students. However, this effect did not occur for physiotherapy-related EBs. In accordance with the cross-sectional study [29], at both time points physiotherapists’ physiotherapy-related EBs were more sophisticated than their medicine-related EBs. Interestingly, the difference between physiotherapy- and medicine-related EBs were quite similar at both time points. Similar developmental trajectories in EBs in different disciplines call into question the assumption that different domain-specific EBs tend to develop asynchronously [33]. Physiotherapy-related and medicine-related EBs emerged equally in both domains. Regarding the theoretical assumption that EBs are discipline-specific but have a domain-general part, we cannot finally conclude whether the development we found relates in particular to a discipline-specific or to a general development of EBs. In future studies both discipline-specific and domain-general EBs should be assessed to address this issue. Based on the data presented here it is unclear whether this result would be transferable to other health domains or to other healthcare professionals. This is an aspect that should also be addressed in future research.

Concerning therapeutic health concepts, the scale of the *bm* and *bps* measure had to be excluded due to psychometric flaws. It is likely that the internal consistency was low because the scale consists of only 5 items and the number of items has an impact on Cronbach’s alpha coefficient [34]. The research question as to whether there is temporal development of the *bm* and *bps* concepts cannot be answered with this study. The generalizability of the results might be reduced by the fact that all of the participating students were recruited from the same school of physiotherapy, whose educational approach was mainly influenced by a bps perspective. Generalizability is also limited because the sample consisted exclusively of physiotherapists trained in Germany; physiotherapy training in Germany differs from training in other countries in that most German physiotherapists attend vocational schools instead of universities. As often is the case in longitudinal studies that take several years to complete, there was a substantial dropout rate that could potentially have biased the study results (in terms of a selection bias). As the same pattern of results was found as in the cross-sectional study, however, it is quite unlikely that the findings in this study result merely from a selection bias.

### Practical implications

As has been shown in several knowledge domains, EBs are an important factor that has an impact on how people process information. It is a key factor for (lifelong) learning, and the temporal development of EBs seems to be an expression of the professionalization of an individual in her or his working field. The findings demonstrate that the development of EBs is not completed at the end of vocational training: it is rather a development that continues even after the transition to professional life. The finding that both physiotherapy-related and medicine-related EBs developed comparably in both domains could be a hint that the temporal development of EBs is discipline-specific and can be interpreted as an indicator of professionalization.

In order to foster the development of EBs during the education process, it might be an option to confront students with close-to-reality learning settings, using for example problem-based learning methods [35], which can facilitate the professionalization process [36]. Another approach could be to develop interprofessional learning settings [37], such as interprofessional videos [38] or interprofessional online learning platforms [39, 40].

## Conclusions

EBs are highly relevant for learning, education, and information processing. Physiotherapy-related and medicine-related EBs developed similarly in both domains. The development of EBs appears to be a continuing development throughout one’s working life. Putting students into realistic learning scenarios already during their physiotherapy training, could be a promising strategy to foster the development of EBs.

## Data Availability

Data are available on request to Martina Bientzle.

## Abbreviations

Bm: Biomedical
Bps: Biopsychological
CAEB: Connotative Aspects of Epistemological Beliefs scale
EBs: Epistemological beliefs
ICD: International Classification of Diseases
ICF: International Classification of Functioning, Disability and Health
